# Periodontal Disease: Linking the Primary Inflammation to Bone Loss

**DOI:** 10.1155/2013/503754

**Published:** 2013-05-23

**Authors:** Adriana Di Benedetto, Isabella Gigante, Silvia Colucci, Maria Grano

**Affiliations:** Department of Basic Medical Sciences, Neurosciences and Sense Organs, Section of Human Anatomy and Histology “R. Amprino”, University of Bari, Piazza Giulio Cesare 11, 70124 Bari, Italy

## Abstract

Periodontal disease (PD), or periodontitis, is defined as a bacterially induced disease of the tooth-supporting (periodontal) tissues. It is characterized by inflammation and bone loss; therefore understanding how they are linked would help to address the most efficacious therapeutic approach. Bacterial infection is the primary etiology but is not sufficient to induce the disease initiation or progression. Indeed, bacteria-derived factors stimulate a local inflammatory reaction and activation of the innate immune system. The innate response involves the recognition of microbial components by host cells, and this event is mediated by toll-like receptors (TLRs) expressed by resident cells and leukocytes. Activation of these cells leads to the release of proinflammatory cytokines and recruitment of phagocytes and lymphocytes. Activation of T and B cells initiates the adaptive immunity with Th1 Th2 Th17 Treg response and antibodies production respectively. In this inflammatory scenario, cytokines involved in bone regulation and maintenance have considerable relevance because tissue destruction is believed to be the consequence of host inflammatory response to the bacterial challenge. In the present review, we summarize host factors including cell populations, cytokines, and mechanisms involved in the destruction of the supporting tissues of the tooth and discuss treatment perspectives based on this knowledge.

## 1. Introduction 

PD is a chronic infectious inflammatory disease that affects periodontium and gradually destroys the tooth-supporting alveolar bone. The periodontium is a supporting structure that surrounds and supports the teeth. It consists of different tissues including the gums, the cementum, the periodontal ligament, and the alveolar supporting bone. 

Periodontal diseases are caused by bacterially derived factors and antigens that stimulate a local inflammatory reaction and activation of the innate immune system [1, 2]. Among the bacterial species that colonize the oral cavity, some of them are associated with periodontitis and are defined as periodontopathogens. The innate host response is initiated by toll-like receptors (TLRs), similar to the protein encoded by the *Drosophila* Toll gene [3]. Toll-like receptors are mainly expressed on cells of the innate immune system [4] but have also been identified in periodontal tissues [5]. Pathogens can invade gingival epithelial cells by binding *β*-1 integrin and replicate, avoiding the host surveillance [6]. Toll-like receptors present on gingival epithelial cells can detect microbial structures highly conserved among pathogens, including lipopolysaccharide (LPS), peptidoglycan, bacterial DNA, double-stranded RNA, and lipoprotein, called pathogen-associated molecular patterns (PAMPs) [7]. Once TLRs present on the surface of resident cells recognize PAMPs, they initiate the activation of several transcription factors including nuclear factor-*κ*B (NF*κ*B) and activator protein 1 (AP-1) through the mitogen-activated protein kinase (MAK) cascade [8, 9]. These in turn activate different innate immunity pathways, including cytokines and chemokines production that recruit nonresident leukocytes to periodontal space. In turn, activated leukocytes, the adaptive immunity cells, secrete proinflammatory cytokines and chemokines in the tissues [10]. It is now accepted that the amplification of this initial local host response (lasting approximately 21 days) results in the propagation of the inflammation and leads to the destruction of soft and mineralized periodontal tissues [11]. The conventional treatment for periodontitis has focused on mechanical removal of bacterial agents, thus reducing infectious challenge and leading to resolution of inflammation and control of PD. However, the standard treatment may not be a sufficient or definitive therapy to result in clinical improvements while requiring a more sophisticated biological approach. Modulation of host response is considered a novel promising therapy and consists in modulating the host defense mechanisms in response to inflammation. To date, the only host modulator drug approved by the United States Food and Drug Administration is SDD (subantimicrobial dose doxycycline). SSD inhibits host-derived matrix metalloproteinases (MMPs), responsible for soft and mineralized tissues degradation, thus resulting in reduced progression of periodontitis [12]. However, an expanding knowledge indicates that several signalings are involved in periodontal tissue destruction and thus, to achieve long-term outcomes, such as prevention of tooth loss, may be necessary to block many pathways. On the other hand, pharmacological host immune modulation may result in adverse side effects, thus requiring a careful monitoring of this approach. Therefore, more detailed insights on cell populations, pathways, and cytokines involved in periodontal pathogenesis would help to address the most efficacious strategies for the care of periodontitis.

## 2. Resident Cells and Innate Immunity

The resident cells involved in the innate host response are many including epithelial cells, gingival and periodontal ligament (PDL) fibroblasts, osteoblast, and dendritic cells [9]. Epithelial cells produce interleukin-8 (IL-8), a neutrophil chemoattractant, which recruits neutrophils migration [13] and increases monocyte adhesion in the blood vessels. Neutrophils that enter the periodontal environment are primed and exhibit increased production of proinflammatory cytokines such as interleukin-1 (IL-1), interleukin-6 (IL-6), and tumor necrosis factor-*α* (TNF-*α*) [14]. These cytokines mediate periodontal tissue destruction by stimulating bone resorption. Monocytes, on the other hand, can differentiate into osteoclasts (OCs) upon different triggers while producing inflammatory cytokines as well; expression of Wnt5a was recently reported in response to lipopolysaccharide (LPS) [15]. 

Dendritic cells (DCs) are encountered once the epithelial barrier is invaded by microorganisms. These cells activate an immune response, either acting as antigen-presenting cells or producing IL-12 and IL-18 that consequently promote interferon-*γ* (IFN-*γ*) secretion by NK cells and later by T cells [16]. Periodontal ligament fibroblasts (PDLFs) and gingival fibroblasts (GF) are the main cells of periodontal soft connective tissue and are accessed as the microorganisms breach the epithelial barrier. They respond through the release of cytokines and degradation molecules. GFs produce TNF-*α*, (IL)-6, (IL)-8, macrophage inflammatory protein (MIP)-1 alpha, and stromal-derived factor (SDF)-1, which are important regulators of inflammatory process and bone metabolism [17–19]. Expression of matrix metalloproteinases (MMPs), laminin-8/9, and laminin-2/4 becomes accentuated [20, 21] in PDLFs; however, these cells also contribute to periodontal inflammation and bone loss via IL-1*β*, IL-6, TNF-*α*, and receptor activator of nuclear factor-*κ*B ligand (RANKL) production and release [19, 22, 23]. Microorganisms can go deeper in the periodontal tissue and reach the surface of alveolar bone. *Porphyromonas gingivalis* has been demonstrated to invade osteoblasts by interacting with integrin *α*5*β*1, inducing actin condensation, JNK pathway activation, and osteoblasts apoptosis [24, 25]. Nonetheless, microbial PAMPs promote the expression of the proosteoclastogenic cytokine RANKL in osteoblasts (OBs), thus promoting osteoclastogenesis [26, 27]. All these events, which represent the initial response to the infection, establish a local inflammation proper of the innate immunity. The inflammatory cytokines produced by resident cells (epithelial cells, GFs, PDLFs, OBs, and DCs) and phagocytes (neutrophils and macrophages) are involved in osteoclastogenesis and are responsible for the alveolar bone loss. 

## 3. Leukocytes and Adaptive Immunity

After this initial response, the infection activates the adaptive immunity process: dendritic cells other than participating to the innate inflammatory response have the ability to capture and present antigens to B and T cells of the acquired immune system [28]. Activated CD4 T-helper cells produce subsets of cytokines which will define phenotypically distinguished immune responses: Th-1 and Th-2 cells, respectively, associate with cellular and humoral immunity [29] and the recently described Th-17 and T regulatory (Treg) cells, which have antagonistic roles as effector and suppressive cells, respectively [10, 30, 31]. B cells are also activated and are transformed into plasma cells, which produce antibodies against bacterial antigens. As a result, tissues affected by periodontitis become colonized with both lymphocytes subtypes, but with a larger proportion of B cells than T cells [32]. Indeed, numerous studies have demonstrated that development of periodontitis involves a switch from a gingivitis lesion, mainly mediated by T cells, to one involving large numbers of B cells and plasma cells [33]. Control of this shift is also mediated by a balance between the Th1 and Th2 subsets of T cells, with chronic periodontitis being mediated by Th2 cells [33]. This inflammatory scenario drives the destruction of connective tissue and alveolar bone. Bone resorbing cells, the osteoclasts, differentiate under the control of RANK/RANKL/OPG system, however a number of cytokines, mainly produced in pathological conditions, have been recently demonstrated to be involved in osteoclastogenesis modulation.

## 4. Cytokines Involved in Bone Loss

### 4.1. RANKL/OPG

RANKL is expressed by osteoblasts and by a number of other cell types, including fibroblasts and T and B lymphocytes. Under pathological conditions, such as those occurring in periodontitis, a dysregulated production of this cytokine occurs. Osteoblasts express TLR1, 2, 4, and 6 and respond to TLR2/6 and TLR2/1 ligands by increasing NF*κ*B activation and RANKL expression levels [34]. Other studies showed that *P. endodontalis* LPS has the ability to promote the expression of RANKL in mouse osteoblasts, and this induction was mainly through the TLR2/4-JNK signaling pathway [27]. Fibroblast expression of RANK-L in physiological conditions is low; however, its expression is accentuated in response to cytolethal toxin from *Aggregatibacter actinomycetemcomitans *and to *Porphyromonas gingivalis* LPS [35, 36]. However, the most abundant source of RANKL in periodontitis is the cells of the immune system. In situ hybridization studies show that high levels of RANKL-specific mRNA transcripts are localized in inflammatory cells, mainly lymphocytes [37]. RANKL-positive lymphocytes are found in the inflammatory connective tissue of the diseased gingival tissue [38], but also circulating T Cells express high levels of RANK-L and spontaneously promote osteoclastogenesis in patients [39]. More precisely the primary source of RANKL in periodontal disease is Th1 or Th17 cells as well as B-cells while Treg cells are shown to attenuate RANKL expression by other activated T cells [40]. Recent studies demonstrate that B cells produce RANKL in response to periodontal pathogen stimulation [41], and that the majority of B cells in periodontal lesions are RANKL+ [42]. In animal models, mice deleted of B cells lack to develop bone loss when infected with *P. gingivalis*, even though B cells are dispensable. Indeed in absence of B cells, T cells still mediate LSP-induced bone loss [43].

The action of RANKL can be blocked by its soluble decoy receptor osteoprotegerin (OPG) which is downregulated in periodontitis, thus resulting in an increased RANKL/OPG ratio. In healthy conditions, OPG is produced by resident periodontal fibroblasts and endothelial cells. Immunohistochemical studies demonstrate significantly lower OPG staining in periodontitis-affected tissue compared to healthy gingival tissue, and gene expression studies report lower OPG expression levels in periodontitis compared to health controls [44]. A study investigates the relative concentrations of RANKL and OPG during the progression of experimental periodontitis induced in mice. A rapid bone loss is observed in the early part of the study, correlating with increased RANKL expression relative to OPG (days 0 to 15). In the last part of the study (days 30 to 60), when the rate of bone loss slowed, RANKL concentration decreases, whereas OPG concentration is high [2]. All the available studies collectively indicate that RANKL increases, whereas OPG decreases in periodontitis; however, no difference is reported in the ratio between patients with mild, moderate, or severe periodontitis [11].

### 4.2. TNF-*α*

Other cytokines as TNF-*α* can synergize with RANK-L in promoting osteoclastogenesis. Further studies show that TNF-*α* activates c-Jun, NF-*κ*B, and calcium signaling leading to NFATc1 activation and thus osteoclast differentiation independent of RANKL in human macrophages [45]. TNF-*α* plays a central role in inflammatory reaction, alveolar bone resorption, and the loss of connective tissue attachment [1, 46]. It is known to be associated in local and systemic inflammation involving bone loss [46]. It is present at high levels in diseased periodontal tissues, where it is positively correlated with RANKL expression [1, 46–48]. Experimental model of periodontitis in primates demonstrates that local injections of TNF-*α* antagonists reduce the appearance of inflammatory cells in the alveolar bone and the formation of bone resorbing osteoclasts. Other studies show spontaneous osteoclast formation and increased bone resorption from circulating PBMCs of periodontitis patients correlating with high levels of TNF-*α* and RANK-L [39, 49]. As a result of the innate immunity response, TNF-*α* is locally produced by neutrophils, which exhibit increased chemotaxis production of proinflammatory cytokines [14]. Macrophages represent an important source of TNF-*α*, that, under dysregulation, contribute to host tissue destruction. After antigenic stimulation, naive CD4+ T cells activate, proliferate, and differentiate into distinct effector cell subsets characterized by their specific cytokine. The Th1 lymphocytes subset is characterized by the secretion of TNF-*α* [50]. In summary, TNF-*α* contributes to periodontal damage by its direct effect on osteoclastogenesis and by amplification of inflammatory immune reactions. Furthermore, in vitro data demonstrate an effect of TNF-*α* not only on osteoclasts, but also on osteoblasts by inhibiting differentiation and bone nodule formation [51].

### 4.3. IL-17

Interleukin-17 (IL-7) is an immune regulatory protein produced by T cells at the inflammation sites. Dysregulation of IL-17 promotes osteoclastogenesis and is associated with bone loss. High levels of IL-17 are found in crevicular fluid of periodontal pockets from patients with periodontitis [52]. Th17 cells characterized as IL-17-producing T-cell subset have been recently identified in chronic PD lesions [53]. Interestingly, IL-17 receptor (IL-17 r) deficient mice display a significant delay in neutrophil recruitment into infected sites [54]. When these mice are exposed to organisms as *P. gingivalis*, They develop increased periodontal bone destruction [55], thus resulting in susceptibility to infection. These data indicate that IL-17 is crucial in the protection against extracellular pathogens and may play a dual role: improving pathogen control and promoting alveolar resorption when released in excessive amounts. IL-17 exerts its osteoclastogenic activity by enhancing RANKL expression on osteoblasts and CD4+ T cells [56]. Furthermore, IL-17 contributes to local inflammation by recruiting and activating immune cells, leading to an abundance of inflammatory cytokines, such as IL-1*β* and TNF-*α*, and RANKL [57].

### 4.4. TRAIL

The TNF-related apoptosis-inducing ligand (TRAIL) was initially known for its apoptotic role in cancer and normal cells [58, 59]; however, recent studies agree on its apoptotic role in osteoblasts and differentiated osteoclasts [60–62]. On the other hand, conflicting results emerge from the literature concerning a role of TRAIL in osteoclastogenesis [63–65]. High levels of TRAIL are found in the serum of PD patients carrying alveolar bone loss [66], and spontaneous osteoclastogenesis is observed in PBMCs cultures from the same patients [39]. Interestingly microorganisms infection is reported to induce osteoblast expression of TRAIL [67]. Addition of TRAIL neutralizing antibodies to PD PBMCs cultures partially rescues spontaneous osteoclastogenesis in a dose-dependent manner. This effect is attributed by the authors to a TRAIL/OPG interaction. In support of this, the addition of RANKL completely rescues the inhibition of osteoclast formation induced by TRAIL neutralizing antibodies [68]. Supporting data demonstrate that the excess of TRAIL over OPG enhances RANKL binding to its receptor RANK by titrating out the inhibitory molecule [69]. Alveolar bone loss in PD could also be determined by a decreased bone formation by osteoblasts. Osteoblasts obtained from alveolar bone fragments of PD patients exhibit a weaker characteristic phenotype compared to control cells and are more sensitive to the apoptotic effect induced by TRAIL [66, 70, 71]. The sensitiveness to TRAIL-induced apoptosis is determined by the ratio between death and decoy receptors. High levels of TRAIL and TRAIL decoy receptors are found in diseased gingival and periodontal tissues, thus favouring apoptosis inhibition in PD and explaining the prolonged survival of inflammatory cells [72].

## 5. Conclusions

**Figure 1:**
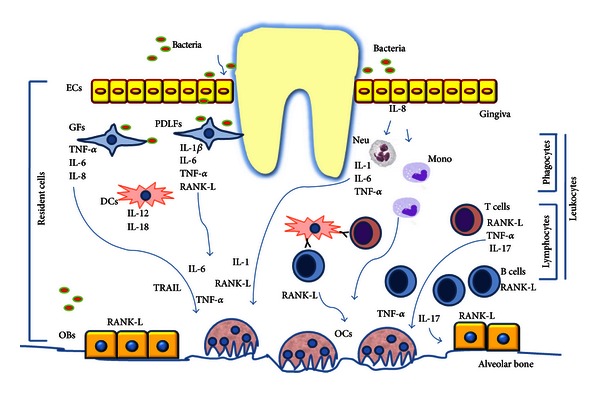
The network of cytokines, released by resident and migrating cells (lymphocytes and phagocytes), involved in periodontal bone resorption. Resident cells including epithelial cells (ECs), gingival fibroblast (GFs), periodontal ligament fibroblasts (PDLFs), osteoblast, and dendritic cells mediate the Innate Immunity. They respond to the bacterial challenge (via TLRs) by producing proinflammatory cytokines and chemokines. ECs produce IL-8, a neutrophil chemoattractant, which recruits neutrophils (neu) and increases monocyte (mono) adhesion. Neu in turn produces IL-1, IL-6, and TNF-*α*, while Mono can differentiate into osteoclasts (OCs). DCs produce IL-12 and IL-18 but also act as antigen-presenting cells for B and T Cells. GFs produce IL-8, TNF-*α*, and IL-6. PDLFs produce IL-1*β*, IL-6, TNF-*α*, and RANKL. Microorganisms can go deeper in the periodontal tissue and reach the surface of alveolar bone, promoting the expression of the proosteoclastogenic cytokine RANKL by osteoblasts (OBs). These inflammatory cytokines are directly (as RANK-L and TNF-*α*) or indirectly involved in osteoclastogenesis and are responsible for the alveolar bone loss. After this initial response (lasting approximately 21 days), activation of T and B cells by antigen-presenting cells initiates the adaptive immunity. As a result, tissues affected by periodontitis become colonized with both lymphocyte subtypes, but with a larger proportion of B cells than T cells. The majority of B cells in periodontal lesions are RANKL+. T cells produce the proosteoclastogenic cytokines RANKL and TNF-*α*, and IL-17 which exerts its osteoclastogenic activity by enhancing RANKL expression on osteoblasts. Furthermore a new role for TRAIL, produced in periodontitis, is emerging in promoting osteoclastogenesis and favoring OBs apoptosis.

The development of periodontitis relies on multiple factors. The disease is of polymicrobial pathogenesis since different types of bacteria are the initiators of the inflammatory process. Innate immunity is the first line of host defense and resistance to infection. Host innate immunity operates through TLRs, which recognize the conserved molecular patterns on pathogenic bacteria. A network of secreted cytokines leads to activation of lymphocytes, but the progression of periodontal lesions is caused by dysregulation of molecules released by specific cell populations. Many of these secreted factors are involved in bone regulation and maintenance, and their imbalance leads to altered periodontal bone remodeling. Thus, enhanced osteoclast activity without increase in bone formation occurs and drives the alveolar bone loss. Mechanical removal of infectious agents in the gingival tissues together with SDD administration as host response modulator is the only current treatment in the care of periodontitis. These approaches attempt to manage the inflammation and control the tissue damage. However, the complexity of pathways involved in the host response drives differences in the clinical manifestation and disease progression, possibly requiring different therapeutic approaches. An important challenge is to understand the different roles of inflammation mediators, their cellular source, their sites of action, and possibly how to control them. In Figure 1, we summarize the network of cytokines, released by resident and migrating cells, involved in periodontal bone resorption. Blocking the activity of proinflammatory cytokines may be a promising therapeutic modality for periodontitis. Some studies have investigated the effect of TNF-*α* and IL-1 antagonists on periodontitis reporting a significant reduction of inflammation and bone resorption, although the studies on TNF-*α* inhibitors produced conflicting results [72]. The RANK/RANKL/OPG axis is a central pathway in the regulation of bone metabolism and is an attractive pharmacological target for the treatment of pathological bone loss. The use of RANKL inhibitors in periodontitis, although limited to animal experimental models, demonstrates a protective effect on alveolar bone resorption [73]. These results encourage to focus on the emerging network of cytokines secreted in PD, some of which are summarized in this review, and to consider them as further therapeutic targets. Nevertheless, it should be remembered that the pathways involved in periodontitis establishment and progression are shared by inflammatory diseases with bone complicance, such as rheumatoid arthritis, multiple myeloma, and cancer. Thus, further insights in the mechanisms linking inflammation to bone loss in periodontitis will also contribute to uncover the impact of immune cells on bone development and maintenance in physiological and pathological conditions. 

## References

[1] Graves DT, Cochran D (2003). The contribution of interleukin-1 and tumor necrosis factor to periodontal tissue destruction. *Journal of Periodontology*.

[2] Garlet GP, Cardoso CR, Silva TA (2006). Cytokine pattern determines the progression of experimental periodontal disease induced by Actinobacillus actinomycetemcomitans through the modulation of MMPs, RANKL, and their physiological inhibitors. *Oral Microbiology and Immunology*.

[3] Hansson GK, Edfeldt K (2005). Toll to be paid at the gateway to the vessel wall. *Arteriosclerosis, Thrombosis, and Vascular Biology*.

[4] Hayashi F, Means TK, Luster AD (2003). Toll-like receptors stimulate human neutrophil function. *Blood*.

[5] Benakanakere M, Kinane DF (2012). Innate cellular responses to the periodontal biofilm. *Frontiers of Oral Biology*.

[6] Bostanci N, Belibasakis GN (2012). *Porphyromonas gingivalis*: an invasive and evasive opportunistic oral pathogen. *FEMS Microbiology Letters*.

[7] Mahanonda R, Pichyangkul S (2007). Toll-like receptors and their role in periodontal health and disease. *Periodontology 2000*.

[8] Hayashi C, Gudino CV, Gibson FC, Genco CA (2010). Pathogen-induced inflammation at sites distant from oral infection: bacterial persistence and induction of cell-specific innate immune inflammatory pathways. *Molecular Oral Microbiology*.

[9] Hans M, Hans VM (2011). Toll-like receptors and their dual role in periodontitis: a review. *Journal of Oral Science*.

[10] Garlet GP (2010). Destructive and protective roles of cytokines in periodontitis: a re-appraisal from host defense and tissue destruction viewpoints. *Journal of Dental Research*.

[11] Cochran DL (2008). Inflammation and bone loss in periodontal disease. *Journal of Periodontology*.

[12] Payne JB, Golub LM (2011). Using tetracyclines to treat osteoporotic/osteopenic bone loss: from the basic science laboratory to the clinic. *Pharmacological Research*.

[13] Han YW, Shi W, Huang GTJ (2000). Interactions between periodontal bacteria and human oral epithelial cells: fusobacterium nucleatum adheres to and invades epithelial cells. *Infection and Immunity*.

[14] Trevani AS, Chorny A, Salamone G (2003). Bacterial DNA activates human neutrophils by a CpG-independent pathway. *European Journal of Immunology*.

[15] Nanbara H, Wara-Aswapati N, Nagasawa T (2012). Modulation of Wnt5a expression by periodontopathic bacteria. *PLoS ONE*.

[16] Tew JG, El Shikh ME, El Sayed RM, Schenkein HA (2012). Dendritic cells, antibodies reactive with oxLDL, and inflammation. *Journal of Dental Research*.

[17] Ekhlassi S, Scruggs LY, Garza T, Montufar-Solis D, Moretti AJ, Klein JR (2008). *Porphyromonas gingivalis* lipopolysaccharide induces tumor necrosis factor-*α* and interleukin-6 secretion, and CCL25 gene expression, in mouse primary gingival cell lines: interleukin-6-driven activation of CCL2. *Journal of Periodontal Research*.

[18] Ara T, Kurata K, Hirai K (2009). Human gingival fibroblasts are critical in sustaining inflammation in periodontal disease. *Journal of Periodontal Research*.

[19] Morandini ACF, Sipert CR, Gasparoto TH (2010). Differential production of macrophage inflammatory protein-1*α*, stromal-derived factor-1, and IL-6 by human cultured periodontal ligament and gingival fibroblasts challenged with lipopolysaccharide from *P. gingivalis*. *Journal of Periodontology*.

[20] Chang YC, Yang SF, Lai CC, Liu JY, Hsieh YS (2002). Regulation of matrix metalloproteinase production by cytokines, pharmacological agents and periodontal pathogens in human periodontal ligament fibroblast cultures. *Journal of Periodontal Research*.

[21] Ohshima M, Yamaguchi Y, Otsuka K, Sato M, Ishikawa M (2006). Laminin expression by human periodontal ligament fibroblasts. *Connective Tissue Research*.

[22] Scheres N, Laine ML, de Vries TJ, Everts V, van Winkelhoff AJ (2010). Gingival and periodontal ligament fibroblasts differ in their inflammatory response to viable *Porphyromonas gingivalis*. *Journal of Periodontal Research*.

[23] Jung IH, Lee DE, Yun JH (2012). Anti-inflammatory effect of (-)-epigallocatechin-3-gallate on *Porphyromonas gingivalis* lipopolysaccharide-stimulated fibroblasts and stem cells derived from human periodontal ligament. *Journal of Periodontal & Implant Science*.

[24] Zhang W, Swearingen EB, Ju J, Rigney T, Tribble GD (2010). *Porphyromonas gingivalis* invades osteoblasts and inhibits bone formation. *Microbes and Infection*.

[25] Zhang W, Ju J, Rigney T, Tribble G (2013). Integrin alpha5beta1-fimbriae binding and actin rearrangement are essential for *Porphyromonas gingivalis* invasion of osteoblasts and subsequent activation of the JNK pathway. *BMC Microbiology*.

[26] Kim M, Jun HK, Choi BK, Cha JH, Yoo YJ (2010). Td92, an outer membrane protein of *Treponema denticola*, induces osteoclastogenesis via prostaglandin-E_2_-mediated RANKL/osteoprotegerin regulation. *Journal of Periodontal Research*.

[27] Tang Y, Sun F, Li X, Zhou Y, Yin S, Zhou X (2011). Porphyromonas endodontalis lipopolysaccharides induce RANKL by mouse osteoblast in a way different from that of *Escherichia coli* lipopolysaccharide. *Journal of Endodontics*.

[28] Cutler CW, Jotwani R (2004). Antigen-presentation and the role of dendritic cells in periodontitis. *Periodontology 2000*.

[29] Murphy KM, Reiner SL (2002). The lineage decisions of helper T cells. *Nature Reviews Immunology*.

[30] Appay V, van Lier RAW, Sallusto F, Roederer M (2008). Phenotype and function of human T lymphocyte subsets: consensus and issues. *Cytometry A*.

[31] Weaver CT, Hatton RD (2009). Interplay between the T_H_17 and T_Reg_ cell lineages: a (co-)evolutionary perspective. *Nature Reviews Immunology*.

[32] Berthelot JM, Le Goff B (2010). Rheumatoid arthritis and periodontal disease. *Joint Bone Spine*.

[33] Ohlrich EJ, Cullinan MP, Seymour GJ (2009). The immunopathogenesis of periodontal disease. *Australian Dental Journal*.

[34] Matsumoto C, Oda T, Yokoyama S (2012). Toll-like receptor 2 heterodimers, TLR2/6 and TLR2/1 induce prostaglandin E production by osteoblasts, osteoclast formation and inflammatory periodontitis. *Biochemical and Biophysical Research Communications*.

[35] Lerner UH (2006). Inflammation-induced bone remodeling in periodontal disease and the influence of post-menopausal osteoporosis. *Journal of Dental Research*.

[36] Park YD, Kim YS, Jung YM (2012). *Porphyromonas gingivalis* lipopolysaccharide regulates interleukin (IL)-17 and IL-23 expression via SIRT1 modulation in human periodontal ligament cells. *Cytokine*.

[37] Liu D, Xu JK, Figliomeni L (2003). Expression of RANKL and OPG mRNA in periodontal disease: possible involvement in bone destruction. *International Journal of Molecular Medicine*.

[38] Lu HK, Chen YL, Chang HC, Li CL, Kuo MYP (2006). Identification of the osteoprotegerin/receptor activator of nuclear factor-kappa B ligand system in gingival crevicular fluid and tissue of patients with chronic periodontitis. *Journal of Periodontal Research*.

[39] Brunetti G, Colucci S, Pignataro P (2005). T cells support osteoclastogenesis in an in vitro model derived from human periodontitis patients. *Journal of Periodontology*.

[40] Belibasakis GN, Bostanci N (2012). The RANKL-OPG system in clinical periodontology. *Journal of Clinical Periodontology*.

[41] Han X, Lin X, Seliger AR, Eastcott J, Kawai T, Taubman MA (2009). Expression of receptor activator of nuclear factor-*κ*B ligand by B cells in response to oral bacteria. *Oral Microbiology and Immunology*.

[42] Kawai T, Matsuyama T, Hosokawa Y (2006). B and T lymphocytes are the primary sources of RANKL in the bone resorptive lesion of periodontal disease. *American Journal of Pathology*.

[43] Yamaguchi M, Ukai T, Kaneko T (2008). T cells are able to promote lipopolysaccharide-induced bone resorption in mice in the absence of B cells. *Journal of Periodontal Research*.

[44] Crotti T, Smith MD, Hirsch R (2003). Receptor activator NF *κ*B ligand (RANKL) and osteoprotegerin (OPG) protein expression in periodontitis. *Journal of Periodontal Research*.

[45] Yarilina A, Xu K, Chen J, Ivashkiv LB (2011). TNF activates calcium-nuclear factor of activated T cells (NFAT)c1 signaling pathways in human macrophages. *Proceedings of the National Academy of Sciences of the United States of America*.

[46] Graves D (2008). Cytokines that promote periodontal tissue destruction. *Journal of Periodontology*.

[47] Ritchlin CT, Haas-Smith SA, Li P, Hicks DG, Schwarz EM (2003). Mechanisms of TNF-*α*- and RANKL-mediated osteoclastogenesis and bone resorption in psoriatic arthritis. *The Journal of Clinical Investigation*.

[48] Garlet GP, Martins W, Fonseca BAL, Ferreira BR, Silva JS (2004). Matrix metalloproteinases, their physiological inhibitors and osteoclast factors are differentially regulated by the cytokine profile in human periodontal disease. *Journal of Clinical Periodontology*.

[49] Tjoa STS, de Vries TJ, Schoenmaker T, Kelder A, Loos BG, Everts V (2008). Formation of osteoclast-like cells from peripheral blood of periodontitis patients occurs without supplementation of macrophage colony-stimulating factor. *Journal of Clinical Periodontology*.

[50] Hernández M, Dutzan N, García-Sesnich J (2011). *Host-Pathogen Interactions in Progressive Chronic Periodontitis, Journal of Dental Research*.

[51] Huang H, Zhao N, Xu X (2011). Dose-specific effects of tumor necrosis factor alpha on osteogenic differentiation of mesenchymal stem cells. *Cell Proliferation*.

[52] Vernal R, Dutzan N, Chaparro A, Puente J, Valenzuela MA, Gamonal J (2005). Levels of interleukin-17 in gingival crevicular fluid and in supernatants of cellular cultures of gingival tissue from patients with chronic periodontitis. *Journal of Clinical Periodontology*.

[53] Cardoso CR, Garlet GP, Crippa GE (2009). Evidence of the presence of T helper type 17 cells in chronic lesions of human periodontal disease. *Oral Microbiology and Immunology*.

[54] Kelly MN, Kolls JK, Happel K (2005). Interteukin-17/interleukin-17 receptor-mediated signaling is important for generation of an optimal polymorphonuclear response against *Toxoplasma gondii* infection. *Infection and Immunity*.

[55] Yu JJ, Ruddy MJ, Wong GC (2007). An essential role for IL-17 in preventing pathogen-initiated bone destruction: recruitment of neutrophils to inflamed bone requires IL-17 receptor-dependent signals. *Blood*.

[56] Boyle WJ, Simonet WS, Lacey DL (2003). Osteoclast differentiation and activation. *Nature*.

[57] Weaver CT, Harrington LE, Mangan PR, Gavrieli M, Murphy KM (2006). Th17: an effector CD4 T cell lineage with regulatory T cell ties. *Immunity*.

[58] Marsters SA, Pitti RA, Sheridan JP, Ashkenazi A (1999). Control of apoptosis signaling by Apo2 ligand. *Recent Progress in Hormone Research*.

[59] Zauli G, Secchiero P (2006). The role of the TRAIL/TRAIL receptors system in hematopoiesis and endothelial cell biology. *Cytokine and Growth Factor Reviews*.

[60] Roux S, Lambert-Comeau P, Saint-Pierre C, Lépine M, Sawan B, Parent JL (2005). Death receptors, Fas and TRAIL receptors, are involved in human osteoclast apoptosis. *Biochemical and Biophysical Research Communications*.

[61] Colucci S, Brunetti G, Cantatore FP (2007). The death receptor DR5 is involved in TRAIL-mediated human osteoclast apoptosis. *Apoptosis*.

[62] Brunetti G, Oranger A, Mori G (2007). Trail is involved in human osteoclast apoptosis. *Annals of the New York Academy of Sciences*.

[63] Secchiero P, Gonelli A, Mirandola P (2002). Tumor necrosis factor-related apoptosis-inducing ligand induces monocytic maturation of leukemic and normal myeloid precursors through a caspase-dependent pathway. *Blood*.

[64] Secchiero P, Melloni E, Heikinheimo M (2004). TRAIL regulates normal erythroid maturation through an ERK-dependent pathway. *Blood*.

[65] Zauli G, Rimondi E, Nicolin V, Melloni E, Celeghini C, Secchiero P (2004). TNF-related apoptosis-inducing ligand (TRAIL) blocks osteoclastic differentiation induced by RANKL plus M-CSF. *Blood*.

[66] Mori G, Brunetti G, Colucci S (2007). Alteration of activity and survival of osteoblasts obtained from human periodontitis patients: role of TRAIL. *Journal of Biological Regulators and Homeostatic Agents*.

[67] Alexander EH, Rivera FA, Marriott I, Anguita J, Bost KL, Hudson MC (2003). *Staphylococcus aureus*—induced tumor necrosis factor—related apoptosis—inducing ligand expression mediates apoptosis and caspase-8 activation in infected osteoblasts. *BMC Microbiology*.

[68] Brunetti G, Oranger A, Mori G (2011). TRAIL effect on osteoclast formation in physiological and pathological conditions. *Frontiers in Bioscience*.

[69] Vitovski S, Phillips JS, Sayers J, Croucher PI (2007). Investigating the interaction between osteoprotegerin and receptor activator of NF-*κ*B or tumor necrosis factor-related apoptosis-inducing ligand: evidence for a pivotal role for osteoprotegerin in regulating two distinct pathways. *The Journal of Biological Chemistry*.

[70] Mori G, Brunetti G, Collucci S (2009). Osteoblast apoptosis in periodontal disease: role of TNF-related apoptosis-inducing ligand. *International Journal of Immunopathology and Pharmacology*.

[71] Brunetti G, Oranger A, Carbone C Osteoblasts display different responsiveness to TRAIL-induced apoptosis during their differentiation process. *Cell Biochemistry and Biophysics*.

[72] Lucas H, Bartold PM, Dharmapatni AA, Holding CA, Haynes DR (2010). Inhibition of apoptosis in periodontitis. *Journal of Dental Research*.

[73] Bartold PM, Cantley MD, Haynes DR (2010). Mechanisms and control of pathologic bone loss in periodontitis. *Periodontology 2000*.

